# Directed Self-Assembly of Cylinder-Forming Block Copolymers Using Pillar Topographic Patterns

**DOI:** 10.3390/polym16070881

**Published:** 2024-03-23

**Authors:** June Huh

**Affiliations:** Department of Chemical and Biological Engineering, Korea University, Seoul 02841, Republic of Korea; junehuh@korea.ac.kr

**Keywords:** block copolymer, directed self-assembly, hole density multiplication, contact hole

## Abstract

We conducted a computational study on the self-assembly behavior of cylinder-forming block copolymers, directed by a guide pattern of hexagonally or tetragonally arrayed pillars, using mesoscale density functional theory simulations. By adjusting the spacing ($L_{p}$) and diameter (*D*) of the pillars in relation to the intrinsic cylinder-to-cylinder distance ($L_{2}$) of the cylinder-forming block copolymer, we investigated the efficiency of multiple-replicating cylinders, generated by the block copolymer, through the pillar-directed self-assembly process. The simulations demonstrated that at specific values of normalized parameters ${\tilde{L}}_{2}=L_{2}/L_{p}$ and $\tilde{D}=D/L_{p}$ coupled with suitable surface fields, triple and quadruple replications are achievable with a hexagonally arrayed pillar pattern, while only double replication is attainable with a tetragonally arrayed pillar pattern. This work, offering an extensive structure map encompassing a wide range of possible parameter spaces, including ${\tilde{L}}_{2}$ and $\tilde{D}$, serves as a valuable guide for designing the contact hole patterning essential in nanoelectronics applications.

## 1. Introduction

Directed self-assembly (DSA), a process involving the structural organization of self-assembling molecules such as block copolymers (BCPs) under the guidance of external processing factors, is a challenging yet crucial technique in various applications, including nanoelectronics. A prime example is the use of chemo-epitaxy or grapho-epitaxy, which involves chemical or topographic patterns to effectively direct BCP self-assembly into desired patterned structures with enhanced pattern precision that is unattainable without the guidance of these prepatterns [1,2,3].

In DSA for nanoelectronics, a meaningful approach, along with line–space patterns [4,5], involves fabricating nanosized contact holes that serve as connection points for electrical signals across different layers or components in semiconductor devices, such as integrated circuits (ICs) [6,7,8,9]. Indeed, numerous recent studies demonstrate that DSA effectively complements extreme ultraviolet lithography (EUV), a premier method in the semiconductor industry [10,11,12]. This synergy, where DSA aids in multiplying, healing, or rectifying EUV patterns, is particularly beneficial given the challenges EUV faces, including stochastic defects, high operational costs, and other limitations [13,14,15,16]. As a specific implementation of a DSA system for contact hole patterning, cylinder-forming BCPs or their blends can be utilized as self-assembling molecules under the guidance of either chemical or topographic patterns. In this case, the pattern transfer from the cylindrical domains of BCPs creates the contact hole patterns. Particularly, when it comes to multiplying contact holes, an effective strategy is to design guide patterns that are congruent with the BCP pattern (i.e., cylinders on a hexagonal array) but have a larger pitch. This larger pitch should ideally match the intrinsic pitch of the BCP pattern, allowing for precise alignment and replication. The pattern multiplication based on this “hexagons within hexagons” strategy also necessitates the appropriate tuning of system or material parameters including the interaction energies between the prepattern and BCP components.

**Figure 1:**
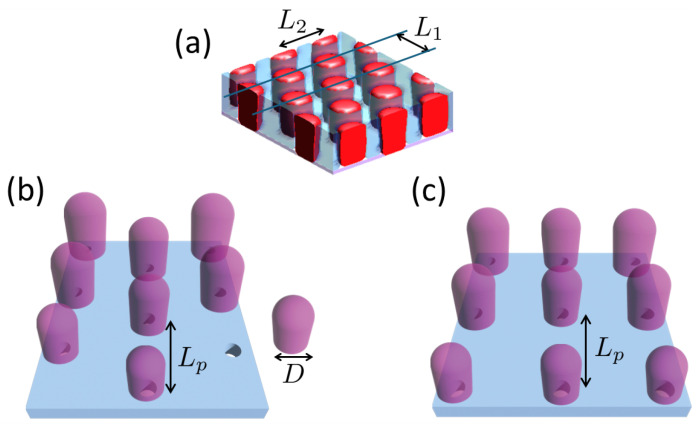
Illustration of (**a**) a cylinder-forming BCP with a natural period $L_{1}=\sqrt{3}L_{2}/2$, (**b**) pillars arranged in a hexagonal array, and (**c**) pillars in a tetragonal array, where $L_{p}$ represents the center-to-center distance between pillars and *D* denotes the diameter of a pillar. The holes beneath the pillars represent the contact holes that were prepatterned.

This work conducts a computational investigation into the self-assembled structures formed by cylinder-forming BCPs under the guidance of topographic pillar patterns. We explore two types of pillar arrays: those arranged in hexagonal and tetragonal configurations. In this DSA system, it is postulated that arrays of contact holes, formed through prepatterning, are capped with hollow cylinders or pillars that serve as a guiding pattern (Figure 1). In semiconductor manufacturing, this type of nanofabrication is indeed feasible; hollow cylinders, typically made from materials such as silicon dioxide (SiO_2_) or similar compounds [17,18,19,20], are strategically placed over contact holes to serve various functions. In particular, one such function is to guide the assembly of material in subsequent layers, which is the specific focus of this study. Upon removal of these pillars after the DSA process, a density multiplication of contact holes can be achieved.

For the model DSA system, we employ a simulation of mesoscale density functional theory simulation based on a modified diffusion equation using Landau–Ginzburg–Cahn–Hilliard (LGCH) theory [21,22]. This simulation was used to investigate the effects of various DSA parameters, including those related to the BCP material (e.g., intrinsic pitch of the BCP) and the prepattern (e.g., pillar-to-pillar distance), on the resulting DSA pattern. This approach enables the construction of DSA structure maps within the associated parameter space.

## 2. Simulation Methods

We model the self-assembly process of a BCP, that is, the phase separation process between monomer types A and B that constitute the BCP, using simulations of mesoscale density functional theory. The key parameter describing phase separation in this mesoscale density functional is the order parameter Ψ(r), which represents the local deviation of the A-monomer fraction ϕ(r) from its average value, i.e., the A-monomer fraction in the BCP *f* at a given position r (thus, Ψ(r)=ϕ(r)−f). The diffusion equation governing the dynamics of Ψ(r) with time *t* is given by

(1) $$\begin{array}{c} \frac{\partial \Psi (r)}{\partial t}=M\nabla^{2}\frac{\partial (F+F_{surf})}{\partial \Psi (r)}+\xi \left(r\right) \end{array}$$

Here, *M* is a mobility constant which we set to unity for simplicity, *F* represents the free energy functional of the BCP, $F_{surf}$ accounts for the free energy associated with the interaction between the BCP component (A and B) and the surface (pillar, substrate, and film top) in contact with the BCP, and ξ represents the thermal noise. The free energy functional, *F*, is approximated using the Landau-type expression

(2) $$\begin{array}{cc} F(\Psi ) & =\int dr\left[-\frac{\tau }{2}\Psi^{2}\left(r\right)+\frac{\mu }{3!}\Psi^{3}\left(r\right)+\frac{\lambda }{4!}\Psi^{4}\left(r\right)+\frac{D}{2}{\left\{\nabla \Psi (r)\right\}}^{2}\right]\\ \; & \;+\frac{b}{2}\int dr_{1}\int dr_{2}G\left(r_{1}-r_{2}\right)\Psi \left(r_{1}\right)\Psi \left(r_{2}\right) \end{array}$$

Here, τ represents a parameter related to the Flory interaction parameter between the A- and B-monomers (χ), functioning similarly to a temperature-like parameter. The parameters μ, λ, and *D* are integral to the chain architecture of the BCP, shaping the conformational contributions. The last term in the expression, characterized by the Green function *G* with a period control parameter *b*, accounts for long-range repulsion, thus penalizing long-wavelength inhomogeneity. The Green function for the Laplacian is given by $\nabla^{2}G\left(r_{1}-r_{2}\right)=-\delta \left(r_{1}-r_{2}\right)$. For a diblock copolymer architecture, the molecular parameters τ, μ, λ, *D*, and *b* are approximated as

(3) $$\begin{array}{cc} \; & \tau =2\left(\chi -\chi_{s}\right)+\frac{3^{1/2}}{Nf^{3/2}{\left(1-f\right)}^{3/2}},\;\mu =\Gamma_{3}/N,\;\lambda =\Gamma_{4}\left(0,0\right)/N,\\ \; & D=\frac{1}{12f(1-f)},\;b=\frac{9}{N^{2}f^{2}{\left(1-f\right)}^{2}} \end{array}$$

where $\chi_{s}$ is the χ at the spinodal, *N* is the number of statistical monomers in a BCP, and $\Gamma_{3}$ and $\Gamma_{4}\left(0,0\right)$ are Leibler vertex functions computed from the monomer correlation functions [23,24,25]. The surface free energy, $F_{surf}$, is expressed as

(4) $$\begin{array}{c} F_{surf}=\sum_{\left\{\alpha \right\}}\int drs_{\alpha }\left(r\right)\Psi \left(r\right) \end{array}$$

Here, $s_{\alpha }\left(r\right)$ is a surface field at the position r in contact with the surface α (where α = *p* for pillar, *s* for substrate, *t* for film top). This field is proportional to the difference in interfacial tension, $\sigma_{\alpha A}-\sigma_{\alpha B}$, where $\sigma_{\alpha A}$ and $\sigma_{\alpha B}$ represent the interfacial tension between surface α and component A and between surface α and component B, respectively.

The geometries of the BCP mesophase and the guide patterns used in the simulation, featuring cylindrical A-domains hexagonally embedded in a B-matrix along with pillars arranged in both hexagonal and tetragonal arrays, are illustrated in Figure 1. The natural period of a cylinder-forming BCP, $L_{1}=\sqrt{3}L_{2}/2$, and the diameter of the pillar, *D*, are the main variables investigated in this study, while the center-to-center distance between adjacent pillars, $L_{p}$, remains constant throughout the investigation. The geometry of a single pillar is composed of a cylindrical region with a height of $0.8L_{p}$ and a capping region formed by a hemisphere with a radius of D/2, where the block copolymer (BCP) fills only the cylindrical region. The fraction, *f*, of the minor component A in the cylinder-forming BCP and the degree of incompatibility, χN, are set to f=0.3 and χN=18, respectively. In our investigation, the variables $L_{2}$ and *D* are explored within the normalized ranges of ${\tilde{L}}_{2}=L_{2}/L_{p}=1/3.0$ to 1/1.7 and $\tilde{D}=D/L_{p}=1/1.9$ to 1/1.3, respectively. These ranges were selected based on their potential to yield interesting DSA structures. For pillars, the meaningful physical sizes are encompassed in the range of $0<\tilde{D}<1$, where $\tilde{D}=1$ signifies the case in which pillars are in contact with their adjacent pillars. Within this range, we focused on $1/1.9<\tilde{D}<1/1.3$, as the DSA systems within this specific range of pillar size exhibited notable results concerning hole density multiplication. Preliminary simulations were also conducted for other pillar sizes ($0<\tilde{D}<1/1.9$ or $1/1.3<\tilde{D}<1$), but these did not yield structures featuring hole density multiplication. Since the natural period $L_{2}$ of a BCP is dependent on *N*, altering *N* serves as a means to adjust ${\tilde{L}}_{2}$, given that $L_{p}$ remains constant. The range of ${\tilde{L}}_{2}$, spanning from 1/3.0 to 1/1.7, is equivalent to *N* ranging from 100 to 320, which roughly translates to the molecular weights from 10 kg/mol to 32 kg/mol, assuming that the monomer has a molecular weight of 100 g/mol. The surface field for the top of the BCP film is set to be neutral between blocks A and B, ($s_{t}=0$), while the surface field for the pillar and substrate can be designed to be attractive to majority B ($s_{p}>0,s_{s}>0$) or neutral.

The diffusion equation Equation (1) is numerically integrated in the discrete space using an explicit method through a numerical algorithm based on the cell dynamics simulation (CDS) approach initially proposed by Oono and Puri [26,27]. This method has been widely utilized for simulating the phase separation phenomena in various multicomponent polymeric systems at mesoscale, including polymer blends and block copolymers, both in bulk and confined spaces, as well as mixtures with particles [28,29,30,31]. All the DSA systems are modeled in an initially disordered state by assigning uniformly distrubuted random numbers between −η and η to the order parameter at each grid point: Ψ(r)∼u(−η,η) where u(−η,η) denotes a uniform distribution ranging from −η to η. The parameter η is set to be 0.1. The surface boundary is treated by a Neumann boundary with vanishing gradient of the order parameter, that is, $\frac{\partial \Psi }{\partial n}{|}_{s}=0$, where *n* represents the direction that is normal for the surface boundary (*s*). This boundary condition implies that there is no flux of the order parameter across the boundary, which corresponds to a reflecting boundary. To numerically update the order parameter at each cell or grid, we utilized $L_{x}\times L_{y}\times L_{z}=96\times 84\times 60$ grids for the DSA systems with hexagonally arrayed pillars and 96×96×60 grids for those with tetragonally arrayed pillars. Here, $L_{\alpha }$ represents the lattice dimension in the α-direction, measured in units of grids. Periodic boundary conditions were applied in the *x* and *y* directions, while reflective boundary conditions were implemented in the *z* direction and at the surface of pillars. Our CDS model, based on the CHC diffusion equation, adheres to a stability condition, formulated as follows: [32]

(5) $$\begin{array}{c} \Delta t<\frac{\Delta x^{4}}{18M-3\left(A-1\right)\Delta x^{2}} \end{array}$$

where Δt is the time step, Δx is the grid spacing, *M* is the diffusivity in Equation (1), and *A* is related to the parameter τ (approximately 1 + τ ) in Equation (3). Given that Δx, M=1, and *A* ranges from 1.1 to 1.25, this condition indicates that the time increment for the stability should be Δt<0.056. All simulations were performed using self-developed code for the CDS algorithm with a time step Δt=0.05.

## 3. Results

In the following, we present the DSA structure maps within the variable space of ${\tilde{L}}_{2}=L_{2}/L_{p}$ and $\tilde{D}=D/L_{p}$ where the geometrical parameters for the cylinder-forming BCP and pillars, $L_{2}$ and *D*, are varied, while the parameter for prepatterned holes, $L_{p}$, remains constant. In practice, this means adjusting the molecular weight of the BCP by controlling *b* through the adjustment of *N* in Equations (2) and (3) as well as the size of pillars (*D*) that are placed over the prepatterned contact holes, thereby keeping $L_{p}$ fixed.

**Figure 2:**
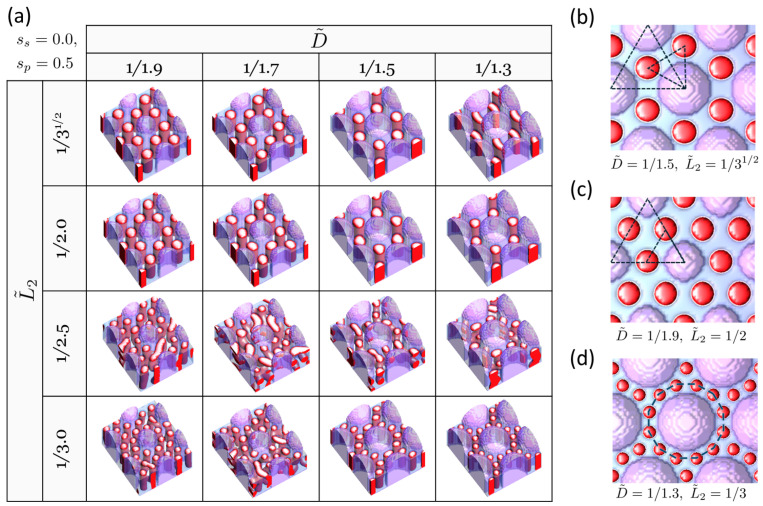
(**a**) DSA structure map in the ($\tilde{D},{\tilde{L}}_{2}$) variable space for cylinder-forming BCP, guided by hexagonally arrayed pillars selective for the majority B block ($s_{p}=0.5$) on a neutral substrate ($s_{s}=0$). The minority A domains of the block copolymer (BCP) are represented by red color, and the minority B domains are depicted by translucent blue, while the pillars are represented by translucent purple color. Notable structures (top views) observed include the following: (**b**) threefold replication of holes, expanding from 4 to 12 holes (H30), (**c**) fourfold replication of holes, expanding from 4 to 16 holes (H0), (**d**) sevenfold replication of holes, expanding from 4 to 28 holes. The dashed triangles in (**b**,**c**) depict the difference in hexagon orientation between the hexagons formed by the pillars and those by the BCP cylinders. The dashed dodecagon in (**d**) represents the BCP cylinders surrounding a pillar.

We begin with the case of hexagonally arrayed pillars. Figure 2a presents the structure map of the BCP self-assembled under the guidance of the hexagonally arrayed pillars that are attractive to the majority B block ($s_{p}=0.5$) on a neutral substrate ($s_{s}=0$). The film thickness *h* of the BCP is set to $\tilde{h}=h/L_{p}=0.8$. This case of surface fields ($s_{p}=0.5$, $s_{s}=0.0$) roughly corresponds to the condition where the pillar is coated with homopolymer B, the same component as the major block B in the cylinder-forming BCP, while the substrate is coated with a random copolymer composed of A and B components. Well-organized cylinder arrays are predominantly found when ${\tilde{L}}_{2}=1/3^{1/2}$ and ${\tilde{L}}_{2}=1/2$, showcasing two distinct hexagonal orientations. Ideally, as shown in Figure 2b,c, a 30° orientation difference between the hexagons formed by the pillars and those by the BCP cylinders (referred to as H30) is expected when ${\tilde{L}}_{2}=1/\sqrt{3}$, whereas a 0° orientation difference (referred to as H0) should be observed when ${\tilde{L}}_{2}=1/2$. In the former case, the number of replicated holes increases from the original 4 prepatterned holes (beneath the pillars) to 12, which includes 8 BCP cylinders in addition to the 4 prepatterned holes, as observed at ($\tilde{D}=1/1.5,{\tilde{L}}_{2}=1/\sqrt{3}$) (Figure 2b). In the latter case, the number increases to 16, consisting of 12 BCP cylinders plus the original 4 prepatterned holes, as identified at ($\tilde{D}=1/1.9,{\tilde{L}}_{2}=1/2$) (Figure 2c). It is noted that both hexagon orientations, H30 and H0, are found not only within their ideal regions (associated with ${\tilde{L}}_{2}=1/\sqrt{3}$ for H30 and ${\tilde{L}}_{2}=1/2$ for H0) but also in regions where ${\tilde{L}}_{2}$ is slightly smaller or larger than these ideal values by compressing or expanding the intrinsic cylinder-to-cylinder distance of the BCP. Furthermore, of interest is the dodecagon structure surrounding a pillar, which results in a sevenfold replication of holes (increasing from 4 to 28 holes), observed at ($\tilde{D}=1/1.3,{\tilde{L}}_{2}=1/3$) (Figure 2d). Unlike the homogeneous tessellation patterns seen with H30 and H0, this case shows a heterogeneous tessellation characterized by the nonuniform spacings between the holes (both pillar-to-cylinder and cylinder-to-cylinder), leading to a pattern that is not a single, uniform tessellation.

**Figure 3:**
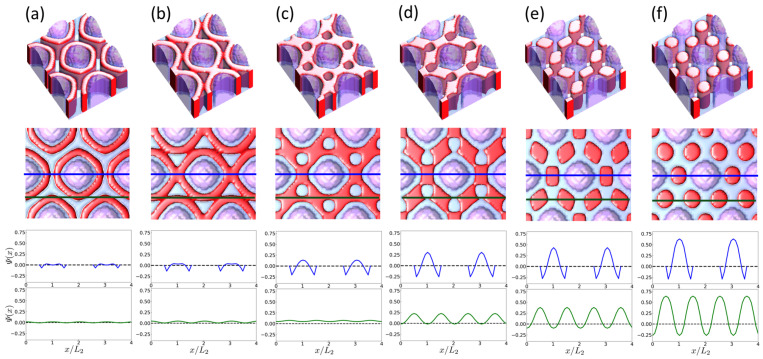
Time evolution of BCP self-assembly guided by hexagonally arrayed pillars with $s_{p}=0.5$ on a neutral substrate ($s_{s}=0$) under the geometric conditions of $\tilde{D}=1/1.9$ and ${\tilde{L}}_{2}=1/2$, depicted in arbitrary time units, *t*. Phases at different times are shown: (**a**) *t* = 100, (**b**) *t* = 300, (**c**) *t* = 600, (**d**) *t* = 1000, (**e**) *t* = 1200, and (**f**) *t* = 2000. For each of (**a**–**f**), the first two images at the top display the angular and top views of the DSA structures, respectively. The minority A domains of the block copolymer (BCP) are represented by red color, and the minority B domains are depicted by translucent blue, while the pillars are represented by translucent purple color. The bottom two plots for each time frame illustrate the order parameter profiles (Ψ(x)=ϕ(x)−f) along the blue and green lines indicated in the top-view images. The dashed line represents the case of a homogeneous state (Ψ(x)=0).

Another interesting aspect is the dynamics of structure evolution, specifically, how the surface fields from pillars arranged in a hexagonal pattern direct the self-assembly of the BCP. Figure 3 illustrates the temporal evolution of the BCP structure shown in Figure 2c, evolving into the H0 structure starting from the early stages of self-assembly. In the initial stages, phase separation starts with AB layering encircling the pillar (Figure 3a). As the layering thickens and the layers start to converge (Figure 3b), cylinders emerge, notably with the B component organized into a hexagonal lattice exhibiting an H30 orientation (Figure 3c). As the self-assembly process progresses, the B domains become interconnected, breaking the continuous A domains into discrete sections, resulting in the inversion of cylinders from B to A (Figure 3d,e), eventually leading to the formation of cylinders composed of the A component with an H0 orientation (Figure 3f).

**Figure 4:**
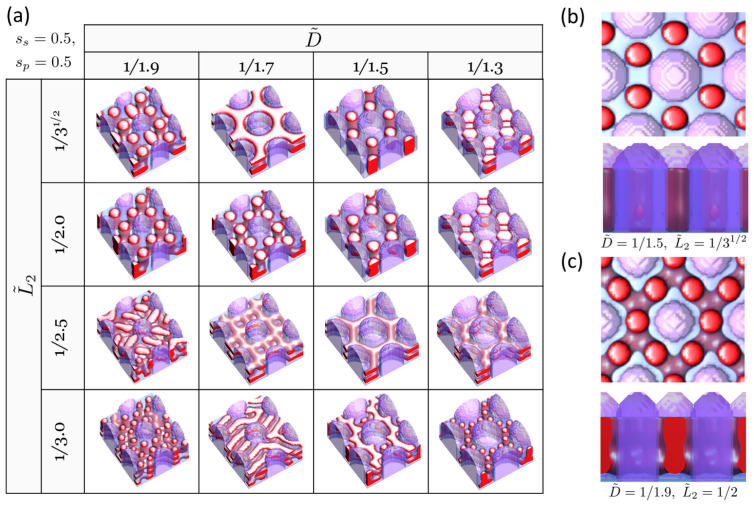
(**a**) DSA structure map in the ($\tilde{D},{\tilde{L}}_{2}$) variable space for cylinder-forming BCP, guided by hexagonally arrayed pillars where both pillars and substrate are selective for the majority B block ($s_{p}=0.5$, $s_{s}=0.5$). The minority A domains of the block copolymer (BCP) are represented by red color, and the minority B domains are depicted by translucent blue, while the pillars are represented by translucent purple color. Notable structures for comparison with Figure 2 include the following: (**b**) top view and side view of DSA structure at ($\tilde{D}=1/1.5,{\tilde{L}}_{2}=1/\sqrt{3}$), (**c**) top view and side view of DSA structure at ($\tilde{D}=1/1.9,{\tilde{L}}_{2}=1/2$).

**Figure 5:**
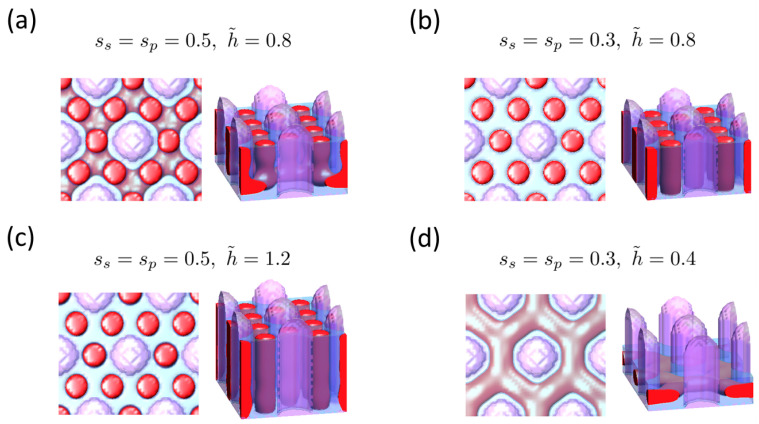
Effect of surface fields and film thickness on the DSA structure guided by hexagonally arrayed pillars with $\tilde{D}=1/1.9,{\tilde{L}}_{2}=1/2$. Displayed structures correspond to the following: (**a**) $s_{s}=s_{p}=0.5$, $\tilde{h}$ = 0.8, (**b**) $s_{s}=s_{p}=0.3$, $\tilde{h}$ = 0.8, (**c**) $s_{s}=s_{p}=0.5$, $\tilde{h}$ = 1.2, and (**d**) $s_{s}=s_{p}=0.3$, $\tilde{h}$ = 0.4. The minority A domains of the block copolymer (BCP) are represented by red color, and the minority B domains are depicted by translucent blue, while the pillars are represented by translucent purple color.

Figure 4a presents the DSA structure map for the same case as Figure 2 but with $s_{p}=0.5$ and $s_{s}=0.5$, i.e., both pillar and substrate are attractive to the major component B. This setup corresponds to both the pillar and the substrate being coated with homopolymer B, which, in terms of the coating process, offers advantages over the previous case ($s_{p}=0.5$, $s_{s}=0.0$), which requires multiple steps for surface modification. Certainly, coating with single-molecular species simplifies the process by enabling completion in a single step. Compared to the neutral substrate case shown in Figure 2, the effect of the surface field from the substrate is obvious, as seen in Figure 4. For instance, at ($\tilde{D}=1/1.5,{\tilde{L}}_{2}=1/\sqrt{3}$), an H30 structure is also formed, but slight modulations from the perfect cylindrical shape are observed (Figure 4b). The substrate effect becomes more pronounced as the area of substrate increases, which corresponds to a decrease in *D*. At ($\tilde{D}=1/1.9,{\tilde{L}}_{2}=1/2$), where H0 is formed in the case of the neutral substrate, the B-attractive substrate induces the formation of connected cylinders that do not come into contact with the substrate due to the formation of the B layer on the substrate (Figure 4c). These imperfections can be mitigated by either reducing the surface fields at both the pillar and the substrate to be weakly attractive to the major B block or by increasing the film thickness, thereby enhancing the pillar wall effect relative to the substrate effect. This is demonstrated in Figure 5, where the impacts of surface fields and film thickness are examined. In practice, a weaker B attraction can be achieved by coating them with a random copolymer composed of components A and B, with a higher fraction of B [33,34].

**Figure 6:**
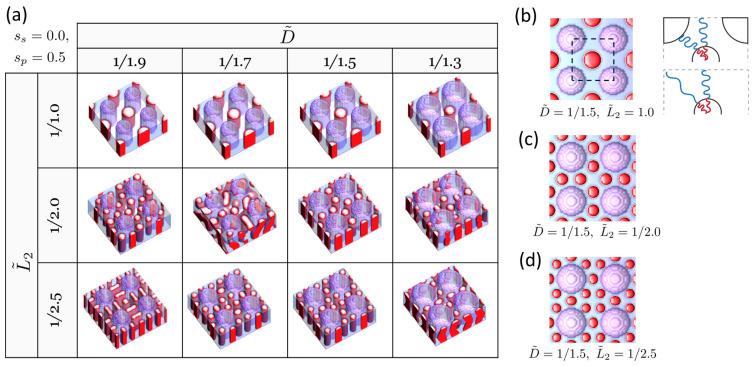
(**a**) DSA structure map in the ($\tilde{D},{\tilde{L}}_{2}$) variable space for cylinder-forming BCP, guided by tetragonally arrayed pillars selective for the majority B block ($s_{p}=0.5$) on a neutral substrate ($s_{s}=0$). The minority A domains of the block copolymer (BCP) are represented by red color, and the minority B domains are depicted by translucent blue, while the pillars are represented by translucent purple color. Notable structures (top views) observed include the following: (**b**) twofold replication of holes, expanding from 4 to 8 holes, (**c**) fivefold replication of holes, expanding from 4 to 20 holes, (**d**) sevenfold replication of holes, expanding from 4 to 28 holes. The schematic in (**b**) illustrates howBCPs, represented by red and blue lines, stretch to fill the space, both in the presence and absence of pillars.

Next, we explore the case involving an alternative guide-pattern geometry: pillars arranged on a tetragonal lattice. This configuration has garnered both academic and practical interest due to the inherent challenges in achieving tetragonal symmetry through self-assembly as compared to the more commonly observed hexagonal symmetry [35,36]. Figure 6a presents the structure map for structures formed by DSA under the guidance of tetragonally arrayed pillars that are selective for the majority B block ($s_{p}=0.5$) on a neutral substrate ($s_{s}=0$). Notably, when $\tilde{L_{2}}=1$, the DSA structure forms a single and uniform tessellation with twofold-replicated holes (increasing from four to eight holes), exhibiting tetragonal symmetry (Figure 6b). One crucial aspect of BCPs, especially when they need to fill a confined space, is their tendency to stretch uniformly as they form mesophases. This underpins the preference for a hexagonal array over a tetragonal array, where BCPs must stretch further to fill the corners in the tetragon compared to the hexagon, even though tetragonally arrayed cylinders have a smaller interfacial area than hexagonally arrayed cylinders. This excessive and undesirable chain stretching of BCPs at the corners of the tetragon can be significantly mitigated by introducing pillars, as illustrated in the schematic in Figure 6b. Another interesting observation includes the formation of heterogeneous tessellations. We identified two instances of such patterns, observed at ($\tilde{D}=1/1.5,{\tilde{L}}_{2}=1/2.0$) and at ($\tilde{D}=1/1.5,{\tilde{L}}_{2}=1/2.5$), where fivefold and sevenfold replications of holes were found, respectively. These notable DSA structures, identified for the tetragonally arrayed pillar case, can similarly be achieved on substrates with pillars coated in a single material (uniform surface fields) by precisely adjusting the film thickness and surface fields, akin to the hexagonally arrayed pillar case.

## 4. Concluding Remarks

In conclusion, this study explores the self-assembly of cylinder-forming BCPs under the guidance of pillar topographic patterns, utilizing mesoscale density functional theory simulations. The investigation encompasses DSA structures of cylinder-forming BCPs across various parameters, including the pitch and diameter of the pillars. Under hexagonally arrayed pillars, we observed that homogeneous tessellations featuring threefold and fourfold hole replications can be realized through the careful adjustment of the pitch and diameter of the pillars, as well as the surface interactions. A previous study employing hexagonally arrayed circles as chemo-patterns for the DSA of cylinder-forming BCPs also reported threefold and fourfold hole multiplication. Similar to the grapho-epitaxial assembly in our work, this multiplication occurs when the distance between circles is $\sqrt{3}$ times and twice larger, respectively, than the natural period $L_{2}$ of the BCP [37]. On the other hand, for tetragonally arrayed pillars, a single instance of homogeneous tessellation was identified, achieving twofold hole replication.

While not all aspects of our simulations could be directly validated by experiments due to the scope and available data, certain key findings show promising alignment with experimental observations conducted by our collaborators at Samsung Electronics [38]. This alignment reinforces the credibility of our simulation approach, particularly in modeling the complex dynamics of DSA systems. The insights from this study provide a theoretical framework for designing DSA systems capable of density multiplication of contact holes, a crucial component in the lithographic processes used for electronic devices.

## Data Availability

Data are contained within the article.
